# Time and Emotion During Lockdown and the Covid-19 Epidemic: Determinants of Our Experience of Time?

**DOI:** 10.3389/fpsyg.2020.616169

**Published:** 2021-01-06

**Authors:** Natalia Martinelli, Sandrine Gil, Clément Belletier, Johann Chevalère, Guillaume Dezecache, Pascal Huguet, Sylvie Droit-Volet

**Affiliations:** ^1^Université Clermont Auvergne, CNRS, LAPSCO, F-63000 Clermont-Ferrand, France; ^2^Université de Poitiers et CNRS, UMR7295 Centre de Recherches sur la Cognition et l’Apprentissage, Poitiers, France

**Keywords:** Covid-19, lockdown, time, emotion, boredom, sleep

## Abstract

To fight against the spread of the coronavirus disease, more than 3 billion people in the world have been confined indoors. Although lockdown is an efficient solution, it has had various psychological consequences that have not yet been fully measured. During the lockdown period in France (April 2020), we conducted two surveys on two large panels of participants to examine how the lockdown disrupted their relationship with time and what this change in their experiences of time means. Numerous questions were asked about the experience of time but also the nature of life during the lockdown: the emotions felt, boredom, the activities performed, sleep quality, and the daily rhythm. The participants also completed a series of self-reported scales used to assess depression, anxiety, and impulsivity. The results showed that time seemed to pass more slowly during the lockdown compared to before. This feeling of a slowing down of time has little to do with living conditions during the lockdown and individual psychological characteristics. The main predictor of this time experience was boredom and partly mediated by the lack of activity. The feeling of being less happy and the presence of sleep disturbance also explained this specific experience of time albeit to a lesser extent.

## Introduction

Due to the Covid-19 crisis, the spring of 2020 was lived in quite exceptional circumstances: more than 3 billion people around the world were confined, i.e., almost half of the world’s population. The lockdown was the only solution deemed to be effective in limiting the spread of the virus and the number of sick people and in keeping hospitals uncluttered. Lockdown is a single solution, but it has different psychological consequences that have not yet been fully measured. One initial consequence is a profound upheaval in our relationship with time. Recent international surveys on the judgment of the passage of time (PoT) during the lockdown suggested that people have experienced a slowing down of time (Cellini et al., 2020; Droit-Volet et al., 2020; Ogden, 2020; Torboli et al., 2020). However, the different factors explaining this change in the experience of time during lockdown were not further analyzed in these initial surveys, which focused on a limited number of factors (e.g., stress, anxiety, social satisfaction, sleep disturbance). It is important to understand the processes underlying the conscious change in our relationship with time during lockdown because this is a familiar and easily accessible feeling that may be indicative of serious psychological problems in the future. The aim of the present study was therefore to further examine people’s experience of time during the lockdown and its different determinants.

Scientists working in the field of time perception have examined the PoT judged retrospectively over a long period of past life—5 or 10 years—(e.g., Wittmann and Lehnhoff, 2005; Friedman and Janssen, 2010; Janssen et al., 2013), but rarely the PoT judged in the present. For judgments of the present time, they have preferred to focus on the human ability to estimate durations based on a neural internal clock system. This has allowed them to avoid the complex question of the conscious judgment of the PoT involving higher-level psychological mechanisms specific to humans (Jonas and Huguet, 2008; Wearden, 2015; Droit-Volet, 2018).

The few studies that have begun to examine the current PoT judgment have focused on several selective factors. For example, some authors have examined the role of time pressure and the number of routines in everyday life self-reported by the participants in their judgment of the speed of the PoT in the current life situation as well as for different past periods (Wittmann et al., 2015; Winkler et al., 2017). Droit-Volet and her colleagues examined the emotion felt (happiness, arousal) and the complexity of the activity carried out and the attention it demanded (Droit-Volet and Wearden, 2015, 2016; Droit-Volet et al., 2017; Droit-Volet, 2019a). Tipples (2018) added the sense of frustration when people are oriented more toward the future than to the present and when the expected event is delayed. Similarly, Wittmann referred to boredom in the specific case of waiting for 7–8 min in relation to individuals’ traits, such as impulsivity (Wittmann et al., 2015; Jokic et al., 2018; Witowska et al., 2020). A general overview of these different studies suggests that PoT judgment is the result of a complex interaction between different kinds of intra- and inter-personal psychological mechanisms. In line with this, Larson, in her model, argued that the PoT judgment depends on the emotion felt, the individual’s cognitive involvement and the stimulus complexity, as well as on the quality of the occupation and the density of the experience (Larson, 2004; Larson and von Eye, 2006). In sum, one might suggest that the experience of time is a simple mirror of the introspective analysis of the self made by individuals as a function of their personal, social, and environmental background (Droit-Volet, 2018; Droit-Volet and Dambrun, 2019).

Accordingly, cognitive and emotional factors are undoubtedly intrinsically interconnected in the experience of time. In addition, the effect of the number of routines but also that of the occupation on the PoT judgment could be mediated by certain attention mechanisms related to the activity performed during the temporal period to be judged. Concerning attention mechanisms, Wearden et al. (2014) found that their participants experienced an acceleration of time when they focused their attention on an activity. Studies on consciousness and mindfulness in meditation practice clearly illustrate this critical role of attention mechanisms in the feeling of time (e.g., Wittmann et al., 2015; Droit-Volet et al., 2018a; Droit-Volet and Dambrun, 2019). The effect of frustration or boredom on the PoT judgment could also be mediated by their associated emotions, namely, anger in the first case and reduced happiness in the second. Several studies have indeed shown the crucial role of emotion in the subjective experience of the PoT (e.g., Droit-Volet and Wearden, 2015, 2016; Droit-Volet, 2019b; Droit-Volet et al., 2019). The more aroused people feel, the more quickly they judge time to pass; the less happy they feel, the more slowly they judge it to pass. Finally, one can assume that activity (and the underlying attention mechanisms) and emotion—both in terms of valence (positive *vs.* negative) and level of arousal (high *vs.* low-arousal)—may be the main predictors of inter-individual differences in time experience during the lockdown and may, therefore, mediate the effect of other factors. In the present study on the experience of time during the lockdown, the participants had to answer a series of self-reported questions assessing both the activities practiced and the emotions felt together with other factors, such as boredom, the focus on the present, or the regularity of the daily rhythm.

What is more, lockdown has been a very unusual life experience in a closed environment in which the usual daily activities are disturbed. It can therefore be argued that living conditions (e.g., size of living space and number of people confined in the same space), sleep quality, which is influenced by the disruption of the daily rhythm, and intra-individual characteristics (e.g., anxiety, depression, and impulsivity) may also influence the experience of time. Indeed, it is easy to imagine that the experience of time will not be the same for people living in a small apartment and those living in a big house, especially when several people live together. As regards individual traits, it has been also shown that people suffering from depression find that time passes slower than other people (e.g., Bschor et al., 2004; Stanghellini et al., 2017; Vogel et al., 2018). In our study, which was conducted on a large sample of participants, we therefore also assessed the living conditions of the participants during lockdown and their psychological traits (i.e., depression, anxiety, happiness trait, impulsivity, and alexithymia).

Given that the lockdown experience is an unprecedented situation that has disrupted people’s everyday activities and thus their inherent feelings, the aim of the present study was to examine (1) the distorted experience of the PoT during compared to before the lockdown and (2) the specific factors underlying this distortion of time. Two large panels of French participants were surveyed in two different studies (i.e., 1332 in Study 1; 1116 in Study 2), both of which were conducted during the particularly strict lockdown imposed on citizens by the authorities in France from 17 March to 11 May 2020 (1–29 April for Study 1 and 24–28 April for Study 2). Using a series of self-reported Likert-type scales, we assessed their subjective time judgments and potentially related factors. We then examined each factor and its statistical relation with the passage-of-time experience, using mediation analyses when they were appropriate.

## Study 1

### Method

#### Participants

A total of 1332 French participants completed the online questionnaire: 1012 women and 320 men (*M_Age_* = 41.05, *SD* = 15.92; *M_Education years_* = 14.85, *SD* = 2.99) (Table 1). They gave their consent after reading a form explaining the guarantee of anonymity and their freedom to stop the survey at any time. The questionnaire was reviewed and approved by the Research Ethics Committee of the University Clermont Auvergne (IRB00011540-2020-31).

**TABLE 1:** Description of participants surveyed in Studies 1 and 2.

	Study 1		Study 2	
	Percentage		Percentage	
**Sex**				
Female	75.98		50.63	
Male	24.02		49.37	
**Marital status**				
Single	37.10		27.90	
No single	62.9		72.10	
**Professional activity^a^**				
Yes	68.92		68.82	
No	31.08		31.18	

	**M**	**SD**	**M**	**SD**

Age	41.05	15.92	45.76	14.967
Education years	14.84	2.99	13.07	2.88
**Lockdown features**				
Area of confinement place (m^2^)	104.17	57.02	97.74	47.15
Confined people number	2.74	1.28	2.6	1.31
Confined duration^b^	4.31	0.67	4.02	0.95
Duration of authorized exits^c^	2.62	1.38	2.81	1.39
Duration of unauthorized exits^c^	1.68	1.66	0.37	1.08
**Psychological scale^d^**				
Happiness (SA-DHS)	64.49	13.58	–	–
Depression (BDI)	4.96	5.07	5.18	6.13
Anxiety (S-STAI)	12.93	4.32	12.92	4.57
Impulsivity (BIS 15)	56.82	17.46	68.72	15.79
Alexithymia (TAS)	47.61	11.85	–	–

*^*a*^Percentage of students: Study 1 = 12.7%, Study 2 = 2.69%.^*b*^Six-point scale: 0 = 0%, 1 = 20%; 2 = 40%, 3 = 60%, 4 = 80%, 5 = 100%. ^*c*^Seven-point scale: 0 = 0, 1 = 5–15 min, 2 = 116*–*30 min, 3 = 35 min–1 h, 4 = 1 h05–2 h, 5 = 2–4 h, 6 = >4 h. ^*d*^Maxi scores: S-UCLA = 40, SA-DHS = 112, BDI = 39, S-STAI = 24, BIS 15 = 135, TAS = 100.*

#### Procedure

The online questionnaire was implemented with LimeSurvey and the data hosted on the local server of the University Clermont Auvergne. Completing the questionnaire took about 40 min since it comprised a large number of questions including the questions of interest, which are described below and are the subject of this article. The questionnaire was distributed via social networks during the French lockdown period between 1 April and 29 April, the lockdown being ordered in France from 17 March to 11 May.

The questionnaire was composed of different demographic questions including questions on the life features of the lockdown: the area of the place of confinement (m^2^), the number of people in the confinement site, the total time spent in the confinement site (0, 20, 40, 60, 80, and 100%), and the average duration per day of authorized and unauthorized exits (0 min, 5–15 min, 16–30 min, 35 min–1 h, 1 h05–2 h, 2–4 h, and >4 h) (Table 1).

With regard to the questions of interest, there were three questions on the experience of time (How do you feel about the speed of the PoT?) each examining three different periods: before the lockdown, during the lockdown, and the present (now). The participants answered on a seven-point scale from 1 (*very slow*) to 7 (*very fast*). A series of questions on the emotion felt were also asked with the same seven-point response scale but going from 1 (*not at all*) to 7 (*a lot*): happiness, anxiety, fear, anger, low arousal (calm/relaxed), and high arousal (stimulated/excited/alert/awake). The participants also answered a question about their feeling of boredom (Do you feel bored?) and whether they were engaging in activities that captured their attention. As for the experience of time, they answered these different questions for three periods: before, during the lockdown, and the present. They were also questioned on their focus on the present, the quality of their sleep (I sleep well) and the regular daily rhythm (the rhythm of my life is regular (waking, meals, and bedtime) but only for the periods before and during the lockdown.

In addition, the participants completed five validated and reliable self-reported scales (Table 1): (1) the Subjective Authentic–Durable Happiness Scale (SA-DHS, Dambrun et al., 2012), which was developed to assess the happiness trait and consists of 16 items rated on a seven-point scale (maximum score = 112); (2) the Beck Depression Inventory (BDI, Beck et al., 1961) (maximum score = 39); (3) the six-item short form of the State-Trait Anxiety Inventory (Spielberger et al., 1983) (S-STAI, Marteau and Bekker, 1992) (maximum score = 24); (4) the 15-item short form of the Barratt Impulsiveness Scale (Barratt, 1959) with a nine-point scale (BIS 15, Spinella, 2007) (total score = 135); and (5) the 20-item Toronto Alexithymia Scale (Bagby et al., 1994), which makes use of a five-point scale (maximum score = 100). These scales were presented in a random order and their reliability was satisfactory (SA-DHS, α = 0.88; BDI, α = 0.82; S-STAI, α = 0.87; BIS 15, α = 0.82; and TAS = 0.84).

#### Statistical Analyses

A series of ANOVAs—with a Bonferroni correction for multiple comparisons applied when necessary—were initially carried out on the time experience with the three periods as within-subject factors: i.e., before the lockdown, during the lockdown, and in the present (now). The same analyses with the three time periods were performed on the other questions of interest (emotion, boredom, activity, sleep, and daily rhythm) with the obvious exception of the questions on the living conditions during the lockdown. Then, as the difference in the judgments during the lockdown and the present was small or non-significant, we decided to calculate a difference index for each of the self-reported responses between before and during the lockdown, and we then transformed this into standardized values (z-scores). A positive value for the time judgment indicated that the participants experienced a slowing down of time during (compared to before) the lockdown, while a negative value indicated a speeding up of time and a null value indicated no change. Correlations between the temporal difference index and the difference index for the other factors were then examined. As previous studies of PoT have focused on a limited number of factors, in a comparative perspective, we decided sparingly to examine the correlation between PoT judgment and factors in each category of factors: lockdown features, psychological traits, emotion, boredom/activity, and sleep/daily rhythm. When several related factors were significantly correlated to time experience, we ran linear regressions by checking the inflation factor (VIF) for the multicollinearity in the regression analyses (Thompson et al., 2017). Finally, for the significant predictors of the time experience, we tested mediation models to investigate the part of variance explained by possible mediating factors. The mediation analyses (5000 bootstraps) were performed using Process version 3 macro (Hayes, 2018) for SPSS. This software was used for all the statistical analyses.

### Results and Discussion

#### Time Experience

**FIGURE 1:**
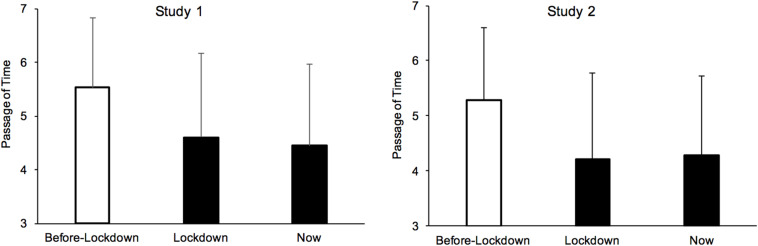
Mean rating of the passage of time for before, during the lockdown and for the present period (Now).

As illustrated in Figure 1, the participants experienced a slowing down of time during the lockdown, both for the lockdown period (*M* = 4.60, *SD* = 1.57) and in the present (*M* = 4.45, *SD* = 1.52), as compared to before the lockdown (*M* = 5.53, *SD* = 1.31) [*F*(1,1331) = 347.17, *p* < 0.001, η^2^*_p_* = 0.21, *F*(1,1331) = 489.46, *p* < 0.001, η^2^*_p_* = 0.27]. Nevertheless, time appeared to pass faster when they considered a longer period of time (lockdown period) than simply the present moment, *F*(1,1331) = 35.13, *p* < 0.001, η^2^*_p_* = 0.02. However, as explained above, the effect size was very small, and we therefore decided to consider only the difference (z-scores) in the time experience between before and during the lockdown period in the subsequent analyses. Marital status (single *vs.* not single) and the fact of having or not having a professional activity did not play a significant role in the difference in the time judgment between the two periods (before *vs.* during the lockdown). Table 2 indicates that the experience of time also did not vary with the participant’s age (*R* = 0.01, *p* = 0.63). There was only a relatively small but significant negative correlation between the time judgment and the level of education (*R* = −0.10, *p* < 0.0001), suggesting that time tended to pass faster during the lockdown for the participants with a higher level of education.

**TABLE 2:** Correlation matrix between age, education, and lockdown features for Studies 1 and 2.

Study 1	Time	1	2	3	4	5	6
1. Age	0.01						
2. Education	−0.10**	–0.05					
3. Confinement area (m2)	0.03	0.10**	0.01				
4. Number of confined people	0.04	−0.23**	–0.03	0.40**			
5. Confined duration	−0.07*	−0.11**	0.07*	0.02	0.07*		
6. Authorized exits duration	0.01	0.11**	0.01	–0.05	−0.12**	−0.37**	
7. Unauthorized exits duration	–0.03	0.17**	0.01	–0.01	−0.06*	−0.28**	0.55**

**Study 2**	**Time**	**1**	**2**	**3**	**4**	**5**	**6**

1. Age	0.01						
2. Education	−0.09**	−0.28**					
3. Living area (m2)	−0.11**	0.11**	0.12**				
4. Number of confined people	0.02	−0.23**	0.10**	0.36**			
5. Confined duration	0.02	0.10**	0.01	0.06*	0.05		
6. Authorized exits duration	0.01	–0.03	0.03	–0.04	–0.03	−0.28**	
7. Unauthorized exits duration	−0.08**	−0.12**	0.02	0.03	0.01	−0.33**	0.16**

*p < 0.05; **p < 0.01.

In conclusion, the lockdown predicted the experience of time—a slowing down of the PoT—without major modulation of people demographic features.

#### Lockdown Features and Time Experience

Table 1 reports the participants’ answers to questions about their living conditions. It appears that the participants complied with the confinement rules, with 95.6 % of individuals staying at home for 80% or more of their time, and the majority of them not exceeding an average of 5–30 min per day of authorized outings (60%) and less than 15 min of unauthorized outings (51%).

The analysis of the relationships between the time experience (the difference between the experience of the PoT before and during the lockdown) and the lockdown conditions of the participants indicated that the changes in time judgments were only weakly or not at all related to the living conditions during lockdown (Table 2). Their time judgments did not vary with the size of the living space (*R* = 0.03, *p* = 0.24) and the number of confined people living together in the same space (*R* = 0.04, *p* = 0.17). The fact of having or not having an outdoor space adjacent to one’s dwelling reduced the feeling of time dragging only very slightly, *F*(1,1330) = 3.94, *p* = 0.05, η^2^*_p_* = 0.003. It seemed that those who spent more time away from home felt time passed a little faster (*R* = −0.07, *p* = 0.01). However, this was only a trend, and the proportion of variance explained was very small.

In sum, our study suggests that life conditions during lockdown did not have a major impact on the experience of time.

#### Psychological Characteristics and Time Experience

The participants’ scores on the different self-reported scales are presented in Table 1 and the correlation between these scores and the experience of time in Table 3. The results in Table 3 indicate that the experience of time had very little or no relation to participants’ scores on the different psychological scales used in our study. Their time experience was related neither to the happiness trait (*R* = 0.01, *p* > 0.05) nor to the level of impulsivity (*R* = 0.01, *p* > 0.05). It tended nevertheless to pass more slowly in the most depressed and anxious participants (*R* = 0.08, *R* = 0.08, *p* < 0.01) and in those who had difficulty expressing their emotions (Alexithymia) (*R* = 0.10, *p* < 0.01). However, when we included these three significant factors (depression, anxiety, and alexithymia) in the same linear regression model, each of them lost their predictive power (*R* = 0.13, *R*^2^ = 0.018, *p* = 0.001; depression, B = 0.025, ES = 0.037, β = 0.026, *t* = 0.69, *p* = 0.49; anxiety, B = 0.058, ES = 0.036, β = 0.057, *t* = 1.59, *p* = 0.11; and alexithymia, B = 0.055, ES = 0.034, β = 0.055, *t* = 1.65, *p* = 0.10).

**TABLE 3:** Correlation matrix between age, education, and scores on the self-reported scales for Studies 1 and 2.

Study 1	Time	1	2	3	4	6
1. Age	0.01					
2. Education	−0.09**	–0.05				
3. Happiness (SA-DHS)	–0.01	0.08**	0.01			
4. Depression (BDI)	0.08**	−0.31**	–0.04	−0.49**		
5. Anxiety (S-STAI)	0.08**	−0.16**	0.01	−0.48**	0.53**	
6. Impulsivity (BIS 15)	0.01	−0.17**	−0.16**	−0.24**	0.33**	
7. Alexithymia (TAS)	0.10**	−0.12**	−0.22**	−0.32**	0.41**	0.36**

**Study 2**	**Time**	**1**	**2**	**3**	**4**	

1. Age	0.01					
2. Education	−0.09**	−0.28**				
3. Depression (BDI)	0.08*	−0.08**	–0.04			
4. Anxiety (S-STAI)	0.13**	–0.05	–0.04	0.49**		
5. Impulsivity (BIS 15)	−0.09**	−0.21**	0.01	0.20**	0.16**	

*p < 0.05; **p < 0.01.

In sum, the scores on the psychological trait scales used in our study did not significantly explain the inter-individual differences in the feeling of the PoT with the lockdown.

#### Emotion and Time Experience

**FIGURE 2:**
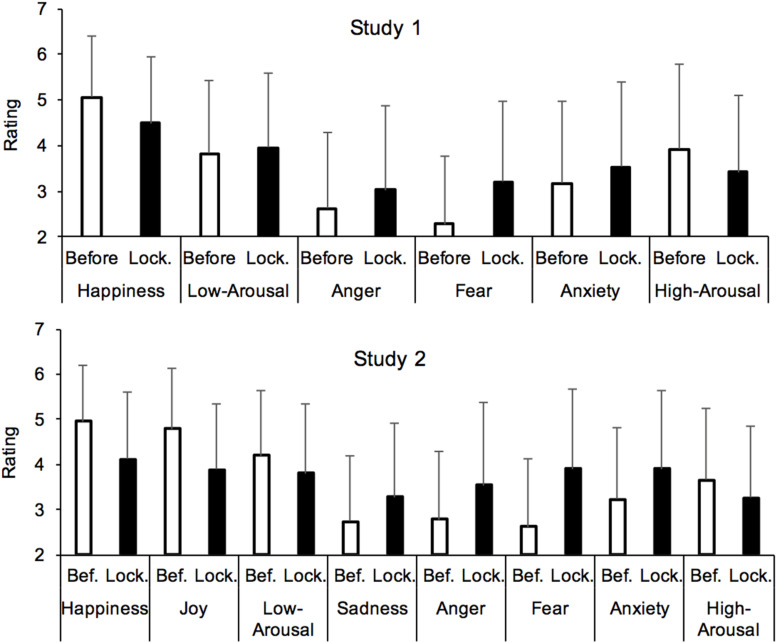
Mean rating of emotions felt before and during the lockdown.

The statistical analyses indicated that differences in the emotional ratings between the lockdown period and the present were either not significant (happiness: *M*_*lockdown*_ = 4.49, *SD*_*lockdown*_ = 1.46, *M*_*present*_ = 4.49, *SD*_*present*_ = 1.55, *F* < 1), or significant but with a small effect size [High-Arousal: *M*_*lockdown*_ = 3.42, *SD*_*lockdown*_ = 1.67, *M*_*present*_ = 3.13, *SD*_*present*_ = 1.64, *F*(1,1331) = 126.35, η^2^*_p_* = 0.09; Low-Arousal, *M*_*lockdown*_ = 3.96, *SD*_*lockdown*_ = 1.63, *M*_*present*_ = 4.21, *SD*_*present*_ = 1.65, *F*(1,1331) = 103.06, η^2^*_p_* = 0.07, all *p* < 0.001], or the ratings were higher for the period of lockdown than for the immediate feeling [Anger: *M*_*lockdown*_ = 3.03, *SD*_*lockdown*_ = 1.83, *M*_*present*_ = 2.27, *SD*_*present*_ = 1.64, *F*(1,1331) = 506.60, η^2^*_p_* = 0.28; Fear, *M*_*lockdown*_ = 3.21, *SD*_*lockdown*_ = 1.74, *M*_*present*_ = 2.54, *SD*_*present*_ = 1.62, *F*(1,1331) = 513.21, η^2^*_p_* = 0.28; Anxiety, *M*_*lockdown*_ = 3.57, *SD*_*lockdown*_ = 1.85, *M*_*present*_ = 2.89, *SD*_*present*_ = 1.77, *F*(1,1331) = 482.44, η^2^*_p_* = 0.26, all *p* < 0.001]. Therefore, we decided to limit the subsequent analyses to the comparison between before and during the lockdown (Figure 2).

The statistical analyses on the emotional ratings comparing the periods before and during the lockdown (Figure 2) showed that the lockdown had a major influence on the participants’ emotions. They did indeed report that the lockdown had an impact both in terms of emotional valence (positive *vs.* negative) and level of arousal (low vs. high-arousal). The participants reported being less aroused during the lockdown than before the lockdown [Low-arousal: 3.96 vs. 3.82 (SD_*before*_ = 1.59), *F*(1,1331) = 9.78, η^2^*_p_* = 0.007, High-arousal: 3.42 vs. 3.92 (*SD*_*before*_ = 1.86), *F*(1,1331) = 122.29, η^2^*_p_* = 0.084]. They also felt less happy during than before the lockdown [4.49 vs. 5.06 (*SD* = 1.35), *F*(1,1331) = 262.31, η^2^*_p_* = 0.17], and reported being more angry, more anxious, and more fearful [angry: 3.03 vs. 2.63 (*SD* = 1.66), *F*(1,1331) = 88.87, η^2^*_p_* = 0.06; anxiety: 3.57 vs. 3.17 (*SD* = 1.81), *F*(1,1331) = 80.34, η^2^*_p_* = 0.06; fear: 3.21 vs. 2.28 (*SD* = 1.47), *F*(1,1331) = 439.72, η^2^*_p_* = 0.25, all *p* < 0.001]. However, among the negative emotions, the effect size was lower for anxiety (η^2^*_p_* = 0.06) and anger (η^2^*_p_* = 0.06) than for fear (η^2^*_p_* = 0.25). Thus, the lockdown was associated with increased feelings of fear and decreased feelings of happiness.

The analyses of correlations (Table 4) between the time experience and the emotional feeling (difference in emotion before and during the lockdown) revealed that the feeling of the PoT was highly sensitive to the emotions felt since the subjective time judgment was significantly related to all the emotions reported (all *p* < 0.01). However, the correlation level was higher for happiness (*R* = 0.25, *p* < 0.0001) than for the other emotions.

**TABLE 4:** Correlation matrix between age, education, emotions, boredom, activity, present focus, sleep quality, and daily rhythm for Studies 1 and 2.

Study 1	Time	1	2	3	4	5	6	7	8	9	10	11	12			
1. Age	0.01															
2. Education	−0.10**	–0.05														
3. Happiness	0.25**	−0.07*	−0.13**													
4. Anxiety	−0.17**	–0.03	0.09**	−0.42**												
5. Fear	−0.12**	0.02	0.09**	−0.32**	0.55**											
6. Anger	−0.14**	0.10**	0.10**	−0.52**	0.38**	0.32**										
7. Low arousal	0.08**	0.03	−0.06*	0.50**	−0.45**	−0.25**	−0.44**									
8. High aoural	0.12**	−0.17**	0.09**	0.01	0.17**	0.10**	0.12**	−0.12**								
9. Boredom	−0.43**	0.24**	0.10**	−0.43**	0.24**	0.19**	0.27**	−0.19**	−0.19**							
10. Activity	0.32**	–0.01	−0.08**	0.35**	−0.15**	−0.10**	−0.20**	0.23**	0.21**	−0.41**						
11. Present-focus	0.01	–0.03	−0.11**	0.22**	−0.13**	−0.07**	−0.15**	0.18**	0.01	−0.12**	0.08**					
12. Sleep	0.09**	−0.10**	–0.03	0.35**	−0.30**	−0.20**	−0.29**	0.36**	−0.11**	−0.17**	0.15**	0.11**				
13. Rhythm	0.10**	−0.13**	–0.04	0.23**	−0.11**	−0.14**	−0.17**	0.17**	0.09**	−0.22**	0.19**	0.09**	0.25**			

**Study 2**	**Time**	**1**	**2**	**3**	**4**	**5**	**6**	**7**	**8**	**9**	**10**	**11**	**12**	**13**	**14**	**15**

1. Age	0.01															
2. Education	−0.09**	−0.28**														
3. Happiness	0.39**	0.12**	−0.09**													
4. Joy	0.36**	0.09**	–0.05	0.66**												
5. Sadness	−0.29**	–0.05	0.03	−0.53**	−0.48**											
6. Anxiety	−0.29**	−0.10**	0.02	−0.42**	−0.41**	0.58**										
7. Fear	−0.22**	−0.14**	0.03	−0.35**	−0.38**	0.48**	0.61**									
8. Anger	−0.31**	−0.14**	0.10**	−0.40**	−0.42**	0.47**	0.49**	0.45**								
9. Low arousal	0.23**	0.14**	−0.07*	0.46**	0.45**	−0.42**	−0.44**	−0.35**	−0.37**							
10. High aoural	0.15**	−0.06*	0.04	0.15**	0.20**	–0.02	–0.02	–0.01	–0.04	0.05						
11. Boredom	−0.45**	0.02	0.01	−0.44**	−0.41**	0.43**	0.36**	0.33**	0.34**	−0.27**	−0.17**					
12. Activity	0.27**	0.01	0.01	0.35**	0.32**	−0.26**	−0.24**	−0.16**	−0.21**	0.23**	0.20**	−0.33**				
13. Present-focus	0.02	0.01	–0.01	–0.01	–0.02	0.04	0.04	0.06	0.04	0.01	0.01	0.01	0.04			
14. Time free	0.06*	0.16**	−0.11**	0.21**	0.15**	−0.09**	−0.14**	–0.04	−0.15**	0.22**	–0.03	–0.02	0.27**	–0.03		
15. Sleep	0.32**	–0.01	–0.05	0.36**	0.33**	−0.34**	−0.35**	−0.28**	−0.29**	0.41**	0.06*	−0.28**	0.21**	–0.02	0.07*	
16. Rhythm	0.26**	−0.07*	–0.05	0.26**	0.24**	−0.19**	−0.18**	−0.16**	−0.10**	0.13**	0.08**	−0.26**	0.18**	–0.03	–0.02	0.33**

*p < 0.05; **p < 0.01.

Although to a lesser extent, the negative emotions were also correlated with the experience of time, with time being judged to pass slower as the levels of anxiety, fear, and anger increased (*R* = −0.17, *R* = −0.14, *R* = −0.12, respectively, all *p* < 0.01). However, the linear regression analysis, with these three negative emotions included in the same model, indicated that anxiety [*B* = −0.124, ES = 0.033, β = −0.124, *t* = −3.72, *p* < 0.001, 95% CIs (−0.189, −0.06), VIF = 1.523] and anger [*B* = −0.085, ES = 0.029, β = −0.085, *t* = −2.889, *p* = 0.004, 95% CIs (−0.142, −0.027), VIF = 1.198] were the only reliable predictors of changes in the experience of time. Indeed, the emotion of fear lost its predictive power [*B* = −0.02, ES = 0.033, β = −0.02, *t* = −0.62, *p* = 0.54, 95% CIs (−0.084, 0.044), VIF = 1.458]. As the hierarchical regression analyses indicated, the total proportion of variance explained by anxiety remained small (*R* = 0.17, *R*^2^ = 0.028) and adding anger to the model increased the proportion of variance explained (*R* = 0.186, *R*^2^ = 0.034) only very little (Δ < 0.01).

The participants, who indicated a decrease in the level of arousal due to the lockdown, also expressed a slowing down of time (low-arousal, *R* = 0.08, *p* = 0.004, high-arousal, *R* = 0.12, *p* < 0.0001). These two arousal-related factors remained significant predictors of the experience of time when entered together in the same regression model [low-arousal, *B* = 0.094, ES = 0.027, β = 0.094, *t* = 3.457, *p* = 0.001, 95% CIs (0.041, 0.148), VIF = 1.015; high-arousal, *B* = 0.126, ES = 0.027, β = 0.126, *t* = 4.625, *p* < 0.0001, 95% CIs (0.073, 0.18), VIF = 1.015]. However, in the same way as for the negative emotions, the total variance explained in the time judgments remained low (*R* = 0.15, *R*^2^ = 0.022).

In sum, the slowing down of time felt by the participants was mainly linked to their level of happiness, which decreased during the lockdown. In other words, the changes in the time experience were better explained by a decrease in positive emotions than by an increase in negative emotions, suggesting that the participants in this study were not prey to a high level of negative emotion. In addition, among the negative emotions, only anxiety played a significant, although still minor, role in the experience of time during the lockdown.

#### Boredom, Activity, and Time Experience

**FIGURE 3:**
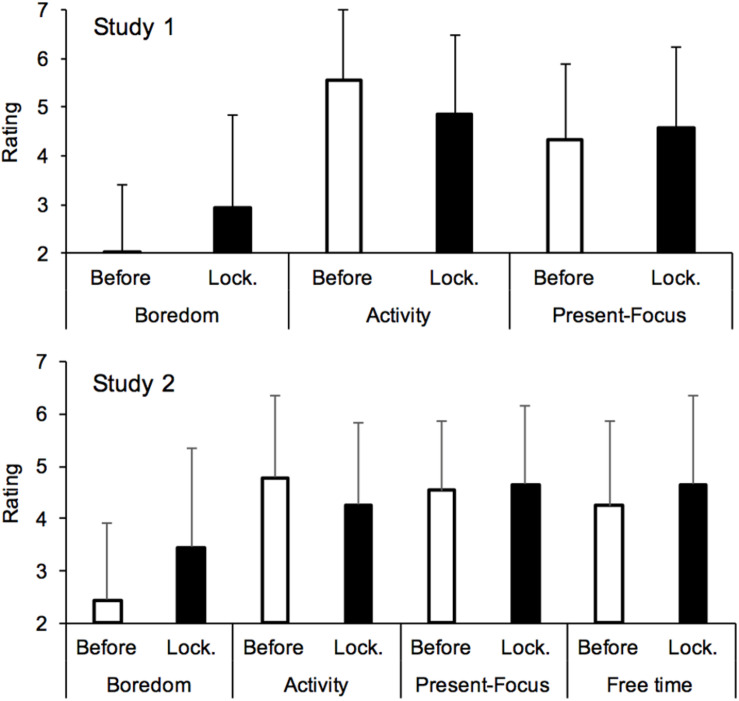
Mean rating for boredom, attention on the activity being performed and being focused on the present for the periods before and during the lockdown.

Being housebound significantly increased the feeling of boredom [*M*_*before*_ = 1.95, *SD*_*before*_ = 1.40, *M*_*lockdown*_ = 2.93, *SD*_*lockdown*_ = 1.89, *F*(1,1331) = 377.11, *p* < 0.001, η^2^*_p_* = 0.22], with fewer attention-demanding activities being carried out [*M*_*before*_ = 5.54, *SD*_*before*_ = 1.47, *M*_*lockdown*_ = 4.84, *SD*_*lockdown*_ = 1.65, *F*(1,1331) = 255.89, *p* < 0.0001, η^2^*_p_* = 0.16] (Figure 3). The boredom level was nevertheless lower in the present (now) (*M*_*present*_ = 2.50, *SD*_*present*_ = 1.89) than when a longer period of lockdown was considered, *F*(1,1331) = 212.63, *p* < 0.001, η^2^*_p_* = 0.14, and when the activity performed in the present was slightly less attention-demanding [*M*_*present*_ = 4.76, *SD*_*present*_ = 1.72, *F*(1,1331) = 4.43, *p* = 0.036, η^2^*_p_* = 0.003]. By contrast, the lockdown had little effect on the participants’ orientation toward the present compared to the past or the future [*M*_*before*_ = 4.32, *SD*_*before*_ = 1.55, *M*_*lockdown*_ = 4.57, *SD*_*lockdown*_ = 4.57, *F*(1,1331) = 32.34, *p* < 0.001, η^2^*_p_* = 0.02].

The analyses of correlations (Table 4) between the changes in the time experience and those relating to boredom and the activity showed that increased boredom and lack of activity were significantly associated with the feeling that time passed more slowly (*R* = −43, *R* = 0.32, *p* < 0.0001). The regression analysis with boredom and activity in the same model revealed that these two factors contributed together to explaining the variance in the experience of time [*R* = 0.46, *R*^2^ = 0.21; *B* = −0.354, ES = 0.027, β = −0.354, *t* = −13.227, *p* < 0.0001, 95% CIs (−0.406, −0.301), VIF = 1.20; *B* = 0.179, ES = 0.027, β = 0.179, *t* = 6.697, *p* < 0.0001, 95% CIs (0.127, 0.231), VIF = 1.20].

In conclusion, these results revealed that the participants’ feeling of time dragging was highly related to the boredom and lack of activity induced by their lockdown at home.

#### Sleep, Daily Rhythm, and Time Experience

**FIGURE 4:**
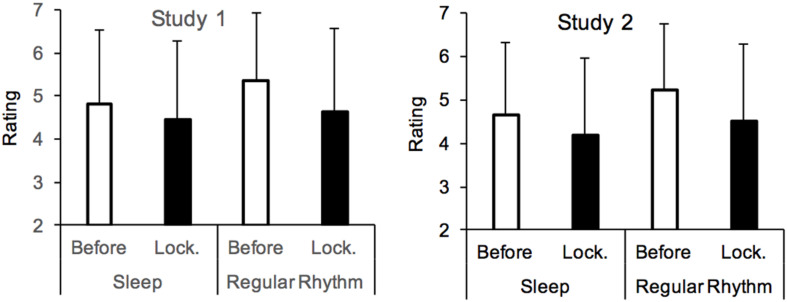
Mean rating for sleep quality and rhythm of life for the periods before and during the lockdown.

The participants also reported that they were sleeping a little less well during than before the lockdown [*M*_*before*_ = 4.81, *SD*_*before*_ = 1.73, *M*_*lockdown*_ = 4.46, *SD*_*lockdown*_ = 1.83, *F*(1,1331) = 70.34, *p* < 0.0001, η^2^*_p_* = 0.05], and that their daily rhythm was less regular [*M*_*before*_ = 5.34, *SD*_*before*_ = 1.71, *M*_*lockdown*_ = 4.65, *SD*_*lockdown*_ = 1.91, *F*(1,1331) = 159.35, *p* = 0.0001, η^2^*_p_* = 0.11] (Figure 4). Those who reported sleeping less well during compared to before the lockdown also felt a slowing down of time (*R* = 0.09, *p* < 0.01) (Table 4). However, they also felt less happy (*R* = 0.35), less calm (*R* = 0.36), more angry (*R* = −0.29), and more anxious (*R* = −30) (all *p* < 0.0001). The regression analysis revealed that the sleep factor was not a reliable predictor of the time experience (B = 0.002, *p* = 0.96), irrespective of whether these emotions (happiness, low-arousal, anger, and fear) were entered together into the same equation or if only one emotion was added to the sleep factor in the regression model (*p* > 0.10). The increase in the irregularity of the daily rhythm with the lockdown was also associated with changes in the time judgment (*R* = 0.10, *p* < 0.01). However, this factor was also highly correlated with, in particular, the happiness and boredom levels (*R* = 0.23, *R* = 0.22, *p* < 0.0001). When these two factors were added to the rhythm factor in the same linear regression model, rhythm also lost its predictive power (*B* = −0.01, *p* = 0.69), while both happiness and boredom remained significant predictors (*p* < 0.01).

Therefore, the results of this study did not suggest direct relationships between the time judgment and the self-reported changes in sleep quality and the regularity of the daily rhythm.

#### Models of Predictors of Time Experience

**FIGURE 5:**
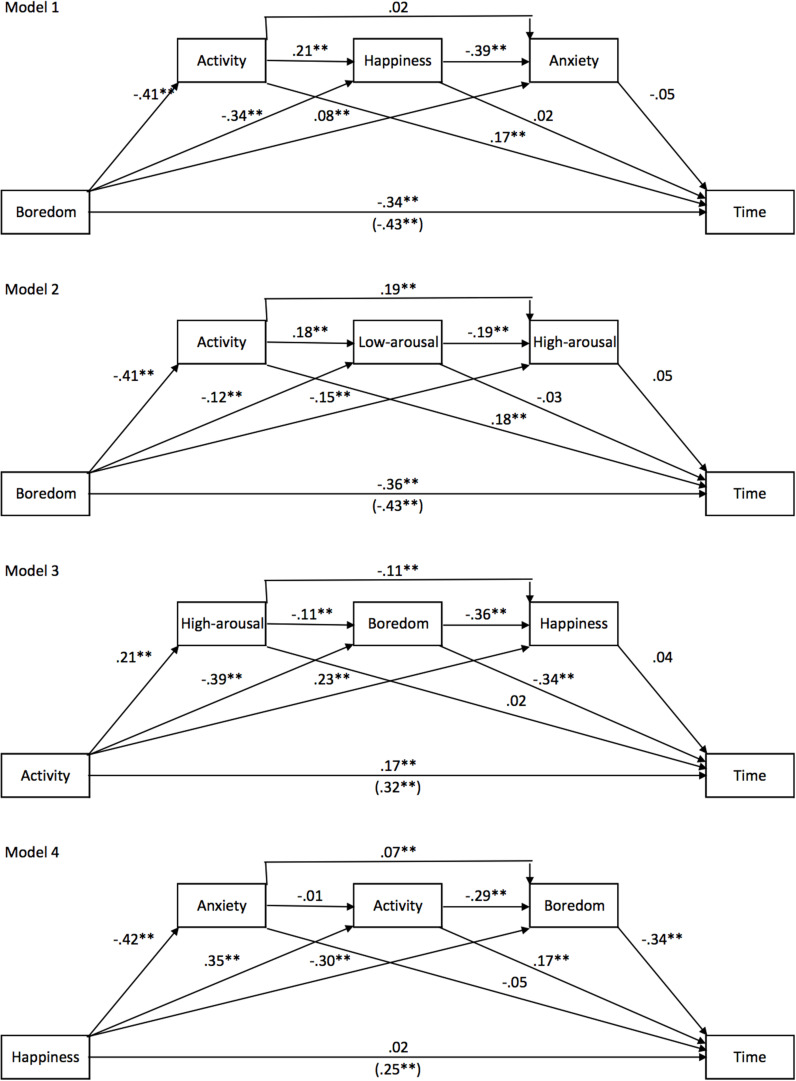
Mediation models for Study 1. Models 1 and 2 show the effect of boredom on time with emotions and activity as mediators. Model 3 shows the effect of activity on time with emotions as mediating factors. Model 4 shows the effect of happiness on time with activity and emotions as mediators.

Our results, therefore, showed that the experience of time was highly sensitive to different self-reported feelings assessed in our survey. However, our statistical analyses suggested that boredom was the main predictor of changes in the time judgment. Boredom was nevertheless correlated with the activity performed during the lockdown as well as the emotion felt (happiness and anxiety) and the level of arousal experienced (low- and high-arousal), and all these factors were significant predictors of subjective time. As boredom is related to both activity and emotion, we tested two models to examine whether the activity and the emotion felt were mediating factors of the effect of boredom on the experience of time (Model 1 and 2, Figure 5). The emotion was characterized by its valence (happiness and anxiety) in the first model and by its level of arousal in the second model (low- and high-arousal). Figure 5 presents these three-way mediation models. The results of these models confirmed the significant direct effect of boredom on the time experience [*B* = −0.336, ES = 0.0284, *t* = −11.808, *p* < 0.001, 95% CIs (−0.3913, −0.2798)]. There was nevertheless an indirect effect of activity [Boredom -> Activity -> Time, *E* = −0.0912, BootSE = 0.0203, BootCIs (−0.1318, −0.0524)], but this only slightly reduced the effect of boredom on the time judgment, accounting for about 20% of the total effect. Adding the emotions of happiness and anxiety and changing their place in the causal relationship did not change the results (*p* > 0.05). The same result was found when arousal level rather than emotional valence was considered as a mediating factor (Model 2). A third mediation model (Model 3) confirmed that boredom mediated the effect of activity on the time judgment for a large proportion with a direct effect of activity of 0.17 (*p* < 0.0001) for a total effect of 0.32 (*p* < 0.0001). Moreover, the direct effect of happiness was no longer significant when boredom and the activity were included in the causal relationship between this emotion and the time judgment (Model 4) [*B* = 0.02, ES = 0.0296, *t* = 0.6855, *p* = 0.49, 95% CIs (−0.037, 0.078)].

In conclusion, our mediation models confirmed that it was mainly the boredom experienced during the lockdown, partly linked to the lack of activity, that led to the feeling that time slowed down during the lockdown compared to before it.

### Discussion

In conclusion, our results suggest that the living conditions during the lockdown and the individuals’ psychological characteristics that we tested did not play a significant role in the experience of time during the lockdown. The contextual effects of the lockdown on the time judgment were thus much stronger than the effects of individual traits. Indeed, the participants’ emotions were deeply disrupted by life in lockdown. The participants felt both less happy and aroused and more anxious, fearful, and angry. However, our results suggest that the feelings of fear and anger were not or only weakly associated with the experience of time during the lockdown. Finally, only the decrease in the feeling of happiness, and to a lesser extent the increase in that of anxiety, influenced the experience of time during the lockdown. However, our statistical analyses indicated that the effect of happiness on the time judgment was mediated by the increase of boredom and the lack of activity during the lockdown. In our study, the major factor that explained the feeling of a slowing down of time during the lockdown period was boredom (itself mediated to a small extent by the lack of activity).

However, in the literature, boredom does not only result from a lack of activity but also has an emotional dimension. It is indeed considered as a low-arousal negative emotion (Eastwood et al., 2012). To verify the role of emotion in the effect of boredom on the feeling that time dragged during the lockdown, we conducted a second study with a new sample of participants. Indeed, the subject sample used in our first study was mainly composed of women and it is well known that both emotion perception and regulation (e.g., Bradley et al., 2001; Gross and John, 2003; Kret and De Gelder, 2012) and activity at home (e.g., child care and housekeeping) (Kamp Dush et al., 2018) differ between women and men. A recent study suggested that women were more stressed at home than men during the lockdown (Droit-Volet et al., 2020). In addition, as happiness and the activity performed played an important role in the time judgment in our first study, we added more specific questions on emotions: one on joy and the other on sadness, which, as positive and negative affects, can be considered as two independent unipolar factors (e.g., Tellegen et al., 1999). Indeed, being less happy does not necessary mean being sadder, i.e., to fall into sadness. We also added a question on the free time available for oneself in order to investigate whether having too much free time could be a source of boredom. Furthermore, we limited the number of psychological scales used to reduce the length of the survey to 30 min instead of 40 min.

## Study 2

### Method

#### Participants and Procedure

The aim of this study was to replicate and extend the results of Study 1 with another sample of participants. The survey was therefore the same and used the same questions though three additional questions were included, i.e., one each for joy, sadness, and the feeling of having free time for oneself. Two self-reported scales were also removed (SA-DHS and TAS) to reduce the survey duration to 30 min. The participants thus only completed the depression (BDI), the anxiety (S-STAI, α = 0.90) and the impulsivity scale (BIS 15, α = 0.79). The statistical analyses conducted were also similar to those used in Study 1.

This new sample was composed of 1116 people: 565 women and 551 men (*M*_*age*_ = 45.76, *SD* = 14.97; *M*_*Education years*_ = 13.4, *SD* = 2.88) (Table 1). They were recruited by a company (Easy panel) from 24 to 28 April (2020). As in Study 1, the participants gave their consent after reading the ethics form, which had been approved by the Research Ethics Committee of the University Clermont Auvergne (IRB00011540-2020-31).

### Results

#### Time Experience

Figure 1 presents the time experience reported by the participants in this new study. The results were similar to those obtained in Study 1, with time judged to be passing more slowly during the lockdown (*M* = 4.22, *SD* = 1.56) and in the present (*M* = 4.28, *SD* = 1.44) than before the lockdown (*M* = 5.28, *SD* = 1.32) [*F*(1,1115) = 349.38, *p* < 0.001, η^2^*_p_* = 0.24; *F*(1,1115) = 358.30, *p* < 0.001, η^2^*_p_* = 0.24], with a slight difference between the time judgment during the lockdown and in the present [*F*(1,1115) = 5.17, *p* < 0.02, η^2^*_p_* = 0.005]. An ANOVA with the lockdown period and participants’ sex as factors showed no main effect of sex (*F* < 1). The interaction between participant’s sex and lockdown period nevertheless reached significance *F*(2,2228) = 3.24, *p* = 0.039, η^2^*_p_* = 0.003. However, this only suggested that time tended to pass faster for the women than for the men before the lockdown [5.39 vs. 5.16, *F*(1,1116) = 7.95, *p* = 0.005, η^2^*_p_* = 0.007], while no sex difference was observed during the lockdown (4.23 vs. 4.22, *F*s < 1). The difference between the subjective judgments of time before and during the lockdown (used in the subsequent analyses) also did not vary with the participants’ age (*R* = 0.01, *p* = 0.70, Table 2). As in Study 1, the feeling of a slowing down of time with the lockdown merely tended to be more pronounced in participants with a lower level of education (*R* = −0.10, *p* = 0.004).

In sum, the feeling that time slowed down during compared to before lockdown was a robust psychological phenomenon observed in both Study 1 and Study 2.

#### Lockdown Features and Time Experience

The results of this second study indicated that the time experience had little to do with the living conditions during lockdown (Table 2). We observed only a small but significant correlation between the living space and the time experience (*R* = −0.11, *p* < 0.0001), suggesting that time seemed to go slightly faster with more living space. In addition, the emotion expressed by the participants did not change with the living space (happiness, *R* = −0.05; sadness, *R* = 0.05; anger = 0.05; fear, *R* = −0.01; anxiety, *R* = −0.01; low-arousal, *R* = −0.004; high-arousal, *R* = −0.06, all *p* < 0.05). A significant but small correlation was observed only with boredom, *R* = 0.06, *p* = 0.047. The impression of time dragging also tended to be reduced in the people who defied the ban on going out (*R* = −0.08, *p* = 0.004). Nevertheless, the levels of correlation remained low.

Our results thus confirm that the experience of time was little affected by the lockdown features.

#### Psychological Characteristics and Time Experience

The analyses of correlations between the subjective experience of time and the scores on the self-reported scales (Table 3) confirmed that the feeling of time fluctuated only slightly, although significantly, with the scores of depression (*R* = 0.08, *p* < 0.01) and anxiety (*R* = 0.13, *p* < 0.01). In Study 2, we also observed a small correlation between the individual level of impulsivity and the judgment of the PoT (*R* = −0.09, *p* < 0.01), a finding not observed in Study 1. No difference between the women and the men was observed for the depression and the impulsivity scores (*F*s < 1), while the women reported being slightly more anxious than the men [13.5 vs. 12.31, *F*(1,1049) = 18.69, *p* < 0.0001, η^2^*_p_* = 0.007]. However, the effect size was not significant. The statistical regression analyses with the depression, anxiety, and impulsivity scores as factors suggested that only anxiety [*B* = 0.126, ES = 0.035, β = 0.127, *t* = 3.59, *p* < 0.0001, 95% CIs (0.057, 0.195), VIF = 1.33], and impulsivity [*B* = −0.128, ES = 0.031, β = 0.128, *t* = −4.07, *p* < 0.001, 95% CIs (−0.189, −0.066), VIF = 1.048] were reliable predictors of the time experience. The assessed level of depression lost its predictive power [*B* = .037, ES = .035, β = .038, *t* = 1.059, *p* = .29, 95% CIs (−.032,.107), VIF = 1.34].

In sum, the more anxious and impulsive the participants were, the longer time seemed to drag on during the lockdown period. However, these relationships were rather weak in terms of shared variance Psychological traits has therefore little effect on PoT judgment relative to the contextual effects of living in lockdown.

#### Emotion and Time Experience

As in Study 1, Study 2 showed that the lockdown had a major influence on affects. The participants indeed reported feeling less positive emotions and more negative emotions. In particular, they felt less happy [*M*_*before*_ = 4.95, *SD*_*before*_ = 1.26, *M*_*lockdown*_ = 4.11, *SD*_*lockdown*_ = 1.48, *F*(1,1115) = 425.29, *p* < 0.001, η^2^*_p_* = 0.28] and joyful [*M*_*before*_ = 4.79, *SD*_*before*_ = 1.33, *M*_*lockdown*_ = 3.88, *SD*_*lockdown*_ = 1.45, *F*(1,1115) = 474.36, *p* < 0.001, η^2^*_p_* = 0.30]. Conversely, they felt sadder [*M*_*before*_ = 2.73, *SD*_*before*_ = 1.46, *M*_*lockdown*_ = 3.28, *SD*_*lockdown*_ = 1.64, *F*(1,1115) = 137.86, *p* < 0.001, η^2^*_p_* = 0.11], more angry [*M*_*before*_ = 2.79, *SD*_*before*_ = 1.49, *M*_*lockdown*_ = 3.54, *SD*_*lockdown*_ = 1.83, *F*(1,1115) = 194.23, *p* > 0.001, η^2^*_p_* = 0.15], more fearful [*M*_*before*_ = 2.62, *SD*_*before*_ = 1.49, *M*_*lockdown*_ = 3.90, *SD*_*lockdown*_ = 1.78, *F*(1,1115) = 623.18, *p* < 0.0001, η^2^*_p_* = 0.36], and more anxious [*M*_*before*_ = 3.22, *SD*_*before*_ = 1.59, *M*_*lockdown*_ = 3.90, *SD*_*lockdown*_ = 1.75, *F*(1,1115) = 198.55, *p* < 0.001, η^2^*_p_* = 0.15]. Their level of arousal also decreased during the lockdown [low-arousal: *M*_*before*_ = 4.20, *SD*_*before*_ = 1.43, *M*_*lockdown*_ = 3.80, *SD*_*lockdown*_ = 1.54, *F*(1,1115) = 64.24, *p* < 0.0001, η^2^*_p_* = 0.05; high-arousal: *M*_*before*_ = 3.66, *SD_*before*_* = 1.58, *M_*lockdown*_* = 3.26, *SD*_*lockdown*_ = 1.59, *F*(1,1115) = 68.90, *p* < 0.001, η^2^*_p_* = 0.06]. The effect of the participants’ sex on the differences between the emotion ratings before and during the lockdown was not significant (*Fs* < 1), with the exception of the feeling of fear, which tended to be higher in women than in men [-0.097 vs. 0.0999, *F*(1,1114) = 10.98, *p* = 0.001, η^2^*_p_* = 0.01].

Table 4, which presents the correlations between the before-during lockdown differences in the experience of time and the emotions felt, confirms the high sensitivity of time judgments to the emotions felt. The time experience varied with all the reported emotions (all *R*s ≥ 0.15, *p* < 0.01). To try to identify the best predictors of the experience of time within each emotion category (positive and negative), we ran two hierarchical linear regression analyses (Table 5), one with the two positive emotions (happiness and joy) and the other with the four negative emotions (sadness, anger, fear, and anxiety). The answers to the questions on emotional arousal (low- and high-arousal) were also included in the same hierarchical regression analysis. These regression analyses revealed that each specific emotion contributed significantly to explaining a proportion of inter-individual differences in the experience of time (all *p*s < 0.01). The only non-significant emotion was the level of fear (β = 0.0001, *t* = −0.001, *p* = 0.99). Therefore, in line with the results of Study 1, the feeling of fear, which increased during the lockdown, did not explain the changes in the experience of time. Furthermore, happiness explained the largest proportion of the variance in the judgment of time, with the decrease in happiness during the lockdown appearing to be the most reliable emotional predictor of the feeling of time slowing down [*B* = 0.39, ES = 0.03, β = 0.39, *t* = −14.06, *p* < 0.001, 95% CIs (0.33, 0.44), VIF = 1, *R*^2^ = 0.15]. The negative emotions (sadness, anger, and anxiety) also played a significant role but the part of variance explained by the various negative emotions was similar (between 8 and 10%) when each emotion was entered first into the equation (sadness, *R* = 0.29, *R*^2^ = 0.084; Anxiety, *R* = 0.29, *R*^2^ = 0.085, Anger, *R* = 308, *R*^2^ = 0.095), with a Δ of 4% when the other negative emotions were added into the equation. Finally, although the third regression model, which included the level of arousal, was significant, it only explained 6–7% of the variance in time judgment (Table 5).

**TABLE 5:** Hierarchical regression analyses on the passage-of-time judgment when the positive emotions, the negative emotions, or the arousal levels were considered in each model.

Model		*B*	ES	β	*t*	*p*	L-CI	U-CI	VIF	*R*^2^
**Positive emotion**										
1	(Constante)	−7.03E-16	0.028				–0.054	0.054		
	Happiness	0.388	0.028	0.388	14.06	0.001	0.334	0.442	1	0.15**
2	(Constante)	−7.51E-16	0.027				–0.054	0.054		
	Happiness	0.268	0.036	0.268	7.40	0.001	0.197	0.339	1.757	
	Joy	0.183	0.036	0.183	5.06	0.001	0.112	0.254	1.757	0.17**
**Negative emotion**										
1	(Constante)	−7.61E-16	0.029				–0.056	0.056		
	Sadness	−0.29	0.029	–0.29	–010.12	0.0001	–0.346	–0.234	1	0.08**
2	(Constante)	−8.04E-16	0.028				–0.055	0.055		
	Sadness	−1.86E-01	0.032	–0.186	–5.84	0.0001	–0.249	–0.124	1.288	
	Anger	−0.22	0.032	–0.22	–6.90	0.0001	–0.283	–0.157	1.288	0.12**
3	(Constante)	−8.18E-16	0.028				–0.055	0.055		
	Sadness	−0.131	0.036	–0.131	–3.68	0.0001	–0.201	–0.061	1.619	
	Anger	−0.185	0.033	–0.185	–5.56	0.0001	–0.25	–0.12	1.42	
	Anxiey	−0.124	0.036	–0.124	–3.44	0.001	–0.195	–0.053	1.661	0.13**
4	(Constante)	−8.18E-16	0.028				–0.055	0.055		
	Sadness	−0.131	0.036	–0.131	–3.64	0.0001	–0.201	–0.06	1.652	
	Anger	−0.185	0.034	–0.185	–5.46	0.0001	–0.252	–0.119	1.466	
	Anxiety	−0.124	0.04	–0.124	–3.12	0.002	–0.202	–0.046	2.013	
	Fear	−3.55E-05	0.037	0.0001	–0.001	0.999	–0.072	0.072	1.721	0.13**
**Arousal**										
1	(Constante)	−7.75E-16	0.029				0.057	0.057		
	Low arousal	0.234	0.029	0.234	8.03	0.0001	0.177	0.291	1	0.06**
2	(Constante)	−7.79E-16	0.029				–0.057	0.057		
	Low arousal	0.228	0.029	0.228	7.88	0.0001	0.171	0.284	1.002	
	High arousal	0.135	0.029	0.135	4.69	0.0001	0.079	0.192	1.002	0.07**

In sum, unlike in Study 1, in Study 2, fear, happiness, sadness, anger, and anxiety seemed to be significant predictors of changes in the judgment of time during the lockdown, although happiness was the best of these. The pattern of the emotions and their links to the time judgment was thus more complex in this second study than in the first one.

#### Boredom, Activity, Present-Focus, Free Time for Oneself, and Time Experience

As shown in Figure 3, boredom was greater during (*M* = 3.45, *SD* = 1.8) than before the lockdown (*M* = 2.45, *SD* = 1.48), *F*(1,1115) = 331.93, *p* < 0.001, η^2^*_p_* = 0.23, with fewer attention-demanding activities being carried out [*M*_*lockdown*_ = 4.26, *SD*_*lockdown*_ = 1.57, *M*_*before*_ = 4.78, *SD*_*before*_ = 1.58, *F*(1,1115) = 103.06, *p* < 0.001, η^2^*_p_* = 0.09]. The participants also considered that they had more time for themselves [*M*_*lockdown*_ = 4.66, *SD*_*lockdown*_ = 1.69, *M*_*before*_ = 4.24, *SD*_*before*_ = 1.63, *F*(1,1115) = 45.88, *p* < 0.001, η^2^*_p_* = 0.04], and that they tended to be more focused on the present [*M*_*lockdown*_ = 4.66, *SD*_*lockdown*_ = 1.51, *M*_*before*_ = 4.54, *SD*_*before*_ = 1.34, *F*(1,1115) = 8.42, *p* = 0.004, η^2^*_p_* = 0.007]. However, as suggested by the effect size of the significant results, only increased boredom represented a major consequence of the lockdown. No effect of participants’ sex was observed on these different dimensions (all *p*s > 0.05), except that the women estimated that they had a little more time for themselves during than before the lockdown than the men did. However, the sex-related difference was also small [−0.065 vs. 0.06, *F*(1,1115) = 4.82, *p* = 0.03, η^2^*_p_* = 0.004].

Table 4 shows the correlations between these factors (boredom, activity, present-focus, free time) and the time experience. The correlation results confirm the close link between the changes in the time experience and the decrease in boredom (*R* = −45, *p* < 0.0001) and the activity performed (*R* = 0.27, *p* < 0.0001). The judgment of time was surprisingly very slightly linked to the feeling of having more free time for oneself (*R* = 0.06, *p* = 0.046) and not at all linked to the fact of being more focused on the present (*R* = 0.02, *p* = 0.53). The regression model with the three significant factors (boredom, activity, and free time) indicated that only boredom and the activity significantly predicted the individual differences in the experience of time [*B* = −0.41, ES = 0.028, β = −0.42, *t* = −14.36, *p* < 0.0001, 95% CIs (−0.46, −0.35), VIF = 1.129; *B* = −0.131, ES = 0.029, β = 0.13, *t* = 4.48, *p* < 0.001, 95% CIs (−0.074, 0.188), VIF = 1.21, respectively]. The fact of having time free for oneself was no longer significant [*B* = 0.018, ES = 0.028, β = 0.018, *t* = 0.66, *p* = 0.51, 95% CIs(−0.036, 0.072), VIF = 1.082]. However, the hierarchical linear regression indicated that boredom explained the greatest proportion of variance in the time judgment, i.e., 20% (*R* = 0.448, *R*^2^ = 0.201), and the Δ was only 0.016% when the activity was added as a factor (*R* = 0.466, *R*^2^ = 0.217).

In sum, this second study confirmed the major role of boredom in the experience of time during the lockdown.

#### Sleep, Daily Rhythm, and Time Experience

The second study confirmed that the quality of sleep was worse during than before the lockdown [*M*_*lockdown*_ = 4.19, *SD*_*lockdown*_ = 1.78, *M*_*before*_ = 4.65, *SD*_*before*_ = 1.68, *F*(1,1115) = 103.32, *p* < 0.001, η^2^*_p_* = 0.09]. The people who reported worse sleep also described time as passing particularly slowly during the lockdown (*R* = 0.32, *p* < 0.0001). The participants also described a less regular rhythm of life during compared to before the lockdown [*M*_*lockdown*_ = 4.52, *SD*_*lockdown*_ = 1.77, *M*_*before*_ = 5.24, *SD*_*before*_ = 1.52, *F*(1,1115) = 181.04, *p* < 0.001, η^2^*_p_* = 0.14]. Furthermore, the more irregular their life was, the more they expressed a slowing down of time (*R* = 0.26, *p* < 0.0001). As in Study 1, the self-reported level of sleep was associated with an increase in the negative emotions and a decrease in the level of arousal (Table 4). However, contrary to Study 1, when we included the sleep factor and the other emotional factors in the same regression model, sleep remained a significant predictor of inter-individual differences in the time judgment (*p* < 0.001).

Therefore, both the decrease in sleep quality and the increase in the irregularity of the life rhythm played a more important role in the time judgment during the lockdown in our second study than was not observed in the first one using another population with the majority of participants completing the survey a little earlier in the lockdown period.

#### The Models of Predictors of the Time Experience

##### Boredom (Figure 6)

**FIGURE 6:**
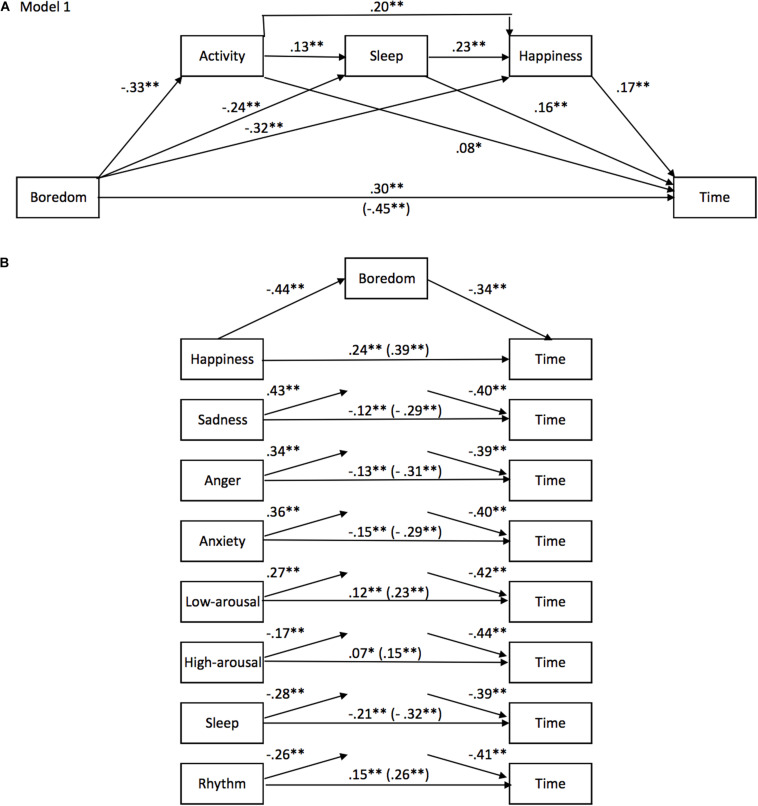
**(A)** Boredom mediation models for Study 2. Model **(A)** shows a mediation model of the effect of boredom on time with activity, sleep and happiness as mediators. Model **(B)** shows mediation models for the effect of each emotion on time with boredom as a mediating factor.

The statistical results of our second study therefore confirmed that boredom was the major factor explaining the feeling of a slowing down of time during the lockdown compared to before. As indicated by the mediation analyses (Figure 6B), boredom mediated the significant effect of each emotion (happiness, sadness anger, and anxiety) on the time judgment as well as that of sleep and daily rhythm. It accounted for between 39 and 48.5% of the total effect of each factor on the time judgment (*p* < 0.01). It also mediated the relationship between the decreased level of arousal and the time judgment (*p* < 0.01) (Figure 6). However, the time judgment was only weakly linked to the levels of arousal (high and low) (see above), and the levels of arousal did not mediate (or did so only to a very small extent) the significant observed effects of the other factors on the time judgment. It was therefore removed from the subsequent analyses. Moreover, the direct effect of boredom on the time judgment remained highly significant even when other mediating factors were entered into the mediation model (Model 1, Figure 6A) [direct effect = −0.31, *SE* = 0.297, *t* = −10.27, *p* < 0.001, 95% CIs (−0.36, −0.25)]. The addition of other factors did not significantly change the direct effect of boredom on the time judgment. The mediation analyses therefore confirmed the main role of boredom in the feeling of a slowing of time during the lockdown.

However, as suggested by the mediation model 1 (Figure 6A), the effect of boredom was significantly mediated by the activity performed during the lockdown [Boredom -> Activity -> Time, Effect = −0.0257, BootSE = 0.0117, BootCIs (−0.0499, −0.0037)], and the quality of sleep [Boredom -> Sleep -> Time, Effect = −0.0384, BootSE = 0.0101, BootCIs (−0.0594, −0.0204)]. In addition, contrary to the results found in Study 1, the mediation analyses revealed that the emotion of happiness was also a significant mediator of the relationship between boredom and the time experience [Boredom -> Happiness -> Time, Effect = −0.0533, BootSE = 0.0133, BootCIs (−0.0818, −0.0288)]. The indirect effect of these factors (activity, sleep, and happiness) accounted for 32% of the total effect of boredom on the time judgment (total of indirect effects = −0.1461, BootSE = 0.0219, BootCIs [−0.1893, −0.1039]). Adding the daily rhythm to the model did not significantly change the mediation percentage [total indirect effects = −0.1573, BootSE = 0.0239, BootCIs (−0.2048, −0.1111)]. Indeed, as described below, the temporal effect of the daily rhythm was mediated both by the sleep difficulties encountered by the participants during the lockdown and their boredom, partly linked to the lack of activity. Similarly, the addition of anxiety and other emotions to the mediation model did not increase the proportion of the explained effect.

In sum, the results of our second study confirmed that boredom mainly accounted for the changes in subjective time during the lockdown, although boredom was mediated only partly by the lack of activity, the emotional state (decreased happiness) and, to a lesser extent, the decrease in sleep quality.

However, as indicated above, and contrary to what was suggested in Study 1, the sensation of time slowing down did not simply result from boredom. The emotional states induced by the lockdown, as well as the quality of sleep and the daily rhythm, partly explained the role of boredom in the subjective slowing down of time. Consequently, despite the significant indirect effect of boredom, the direct effect of other factors remained significant (Figure 6 all *p* < 0.01). We therefore examined the relevance of other factors (emotion, sleep, and daily rhythm) for the time judgment.

##### Emotion (Figure 7)

**FIGURE 7:**
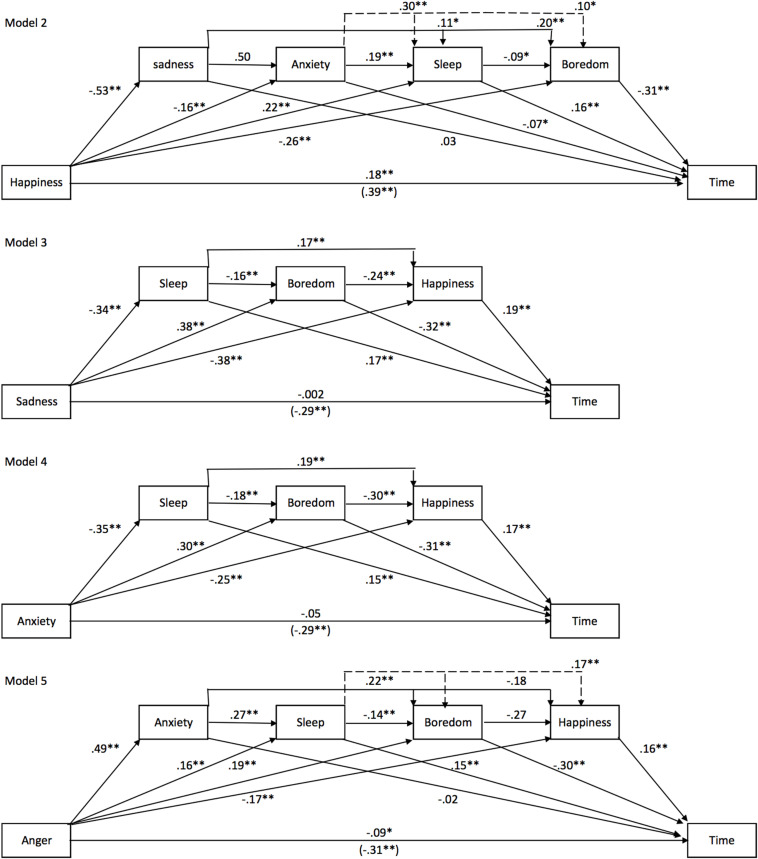
Emotion mediation models for Study 2. Models of mediation for the effect of emotions on time: Model 2, Happiness; Model 3, Sadness; Model 4, Anxiety; Model 5, Anger.

Among the relevant emotion-related factors, the feeling of decreased happiness during the lockdown appeared to be the most reliable predictor of the time experience. Indeed, the effect of other emotional factors on the time judgment was systematically mediated by the decreased happiness (all *p* < 0.0001). However, happiness was also mediated by boredom. We therefore tested a complete mediation model (Model 2, Figure 7), which included sadness, anxiety, sleep quality, and boredom as mediating variables (adding a factor or substituting a factor by another factor did not change or even reduce the percentage of indirect effect). This model indicates that sadness *per se* was not a significant mediator of the effect of happiness on the subjective experience of time [Happiness -> Sadness -> Time, Effect = −0.0164, BootSE = 0.0198 BootCIs (−0.0543, 0.0236)]. In the self-assessments of their feelings during the lockdown, the participants therefore did not directly associate a decrease in their feeling of happiness with an increase in sadness. In the same way as sadness, anxiety was not a significant mediator [Happiness -> Anxiety -> Time, Effect = 0.0105, BootSE = 0.0066 BootCIs (−0.0015, 0.0247)]. Boredom and sleep quality on their own accounted for 46 % of the total effect of decreased happiness on the subjective slowing down of time during the lockdown [i.e., 18% of the total of the indirect effects of model 2, E = 0.2056, BootSE = 0.0285, BootCIs (−0.1506, 0.2617)]. Nevertheless, the direct effect of happiness remained significant (direct effect of happiness = 0.1823, *SE* = 0.0323, *t* = 5.64, *p* < 0.001 95% CIs [0.1190, 0.2457]. In sum, the results of Study 2 showed that decreased happiness was a major emotional factor, which went some way to explaining the participants’ experience of time during the lockdown.

While happiness remained a significant predictor of changes in time judgment during the lockdown, sadness lost its predictive power when sleep difficulties, boredom, and decreased happiness were used as mediating variables [direct effect of sadness = 0.002, *SE* = 0.0317, *t* = 0.0522, *p* = 0.95, 95% CIs (−0.0605, 0.0638)] (Figure 7, model 3). The same results were found for anxiety [direction effect of anxiety = −0.05, *SE* = 0.0296, *t* = −1.81, *p* = 0.07, 95% CIs (−0.1116, 0.0044)] (Figure 7, model 4). With a similar model (Figure 7, model 5), anger kept its power to predict the time judgment but at a low level, i.e., 29% of the total effect [direct effect of anger = −0.0911, *SE* = 0.0306, *t* = −2.98, *p* = 0.003, 95% CIs (−0.1511, −0.0311)]. Furthermore, anxiety did not mediate the effect of anger on the time judgment [Anger -> Anxiety -> Time, Effect = −0.0106, BootSE = 0.0188, BootCIs (−0.0470, 0.0261)], or indeed that of happiness or of another emotion once boredom, in particular, was included in the mediation model. Therefore, the increase in both the sadness and the anxiety induced by the lockdown did not directly contribute to changes in the time judgment during the lockdown. By contrast, anger contributed to it, albeit at a very low level.

In sum, negative emotions (sadness, anger, fear, and anxiety) experienced during the lockdown had not or few effects on the experience of a slowing of time during lockdown. Only a decrease in the level of happiness played an important role, mediated in part by boredom and sleep disruption caused by the lockdown.

##### Sleep, daily rhythm (Figure 8)

**FIGURE 8:**
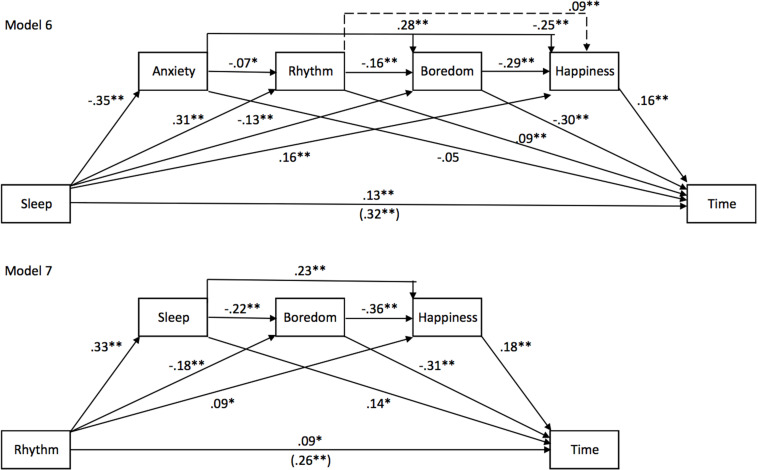
Sleep and daily-rhythm mediation models for Study 2. Model 6 shows the effect of sleep and model 7 that of rhythm of life on time.

Finally, the sleep difficulties encountered by the participants during the lockdown played an important role in the experience of time during this period. The direct effect of this sleep-related factor was preserved in the different mediation models (Figure 8, model 6) [direct effect of sleep = 0.1299, *SE* = 0.0291, *t* = 4.46, *p* < 0.001, 95% CIs (0.0728, 0.1870)], even though its predictive level was lower than those observed for boredom and happiness. As observed above, in the mediation model tested, anxiety once again did not play a significant role in the sleep effect on the time judgment [Sleep -> Anxiety -> Time, Effect = −0.0189, BootSE = 0.0120, BootCIs (−0.0041, 0.0434)]. However, the daily rhythm, boredom, and decreased happiness were significant mediators of the sleep effect on the feeling of time dragging during the lockdown [Sleep -> Rhythm -> Time, Effect = −0.0284, BootSE = 0.0119, BootCIs (0.0062, 0.0533); Sleep -> Boredom -> Time, Effect = 0.0383, BootSE = 0.0121, BootCIs (0.0167, 0.0636); Sleep -> Happiness -> Time, Effect = 0.0265, BootSE = 0.0086, BootCIs (0.0114, 0.0451)], with the result that their combined effects accounted for 59.62% of the total effect of sleep quality on the time judgment (model 6) [total of indirect effects = 0.1918, BootSE = 0.0243, BootCIs (0.1446, 0.2412), *t* = 4.46, *p* < 0.001, 95% CIs (0.0728, 0.1870)]. Nevertheless, as noted immediately above, the direct effect of the sleep factor remained significant.

Although the daily rhythm also influenced the assessment of the PoT during the lockdown, its effect was largely mediated by the sleep difficulties encountered by the participants associated with their boredom and their decreased happiness. A total of 65.38% of the effect of the daily rhythm on the time judgment was indeed mediated by these factors [total indirect effects = 0.17, BootSE = 0.0209, BootCIs (0.1318, 0.2127)]. However, the unstructured rhythm of life during the lockdown continued to account for a small part of the inter-individual differences in the time judgment, as indicated by the significant direct effect of the daily rhythm [direct effect of the daily rhythm = 0.09, *SE* = 0.0277, *t* = 3.32, *p* = 0.001, 95% CIs (0.0376, 0.1462)]. The addition of the activity as a mediating variable did not change the results of the equation.

In sum, difficulty sleeping and, to a lesser extent, the irregularity of the daily rhythm partly explained the feeling of time dragging during the lockdown.

In conclusion, the different mediation models conducted in Study 2 clearly indicated that three factors had indirect and direct effects on the feeling of a slowing down of time during lockdown compared to before, which were boredom, decreased happiness, and sleep difficulties, although the main factor was boredom.

## General Discussion

During the exceptional period of the Covid-19 epidemic, when people were forced to remain confined to their homes, we conducted two surveys in France with two large samples of over 1000 participants each. This makes it possible to verify the replication of the results and their robustness from one study to the next, in which a different sample was used. Both samples were tested over the same time period (i.e., April 2020), although the second survey was completed later (between 24 and 28 April for Study 2 and between 1 and 29 April for Study 1). It would also have been interesting to examine changes in participants’ responses over the entire lockdown period and/or at two distinct moments of the lockdown. In addition, although the results were fairly straightforward in showing the major role of boredom in time judgment, the use of mediation models in cross-sectional data should be used with caution (O’Laughlin et al., 2018). An interest of our study lies in having questioned the participants on their feeling and behaviors before the lockdown. It would have been, however, preferable, although impossible here, to have their initial ratings outside the lockdown period rather than retrospective responses. Interviewing the same people at another time period would therefore be important to confirm our results.

The results of our two surveys nevertheless clearly showed that the lockdown greatly disrupted the participants’ life events and their emotions. The main consequence of these changes was that the participants’ relationship to time was altered: Time seemed to pass far more slowly compared to before the lockdown. This feeling of a slowing down of time with the lockdown is a robust psychological phenomenon reported in all the international studies on the time judgment conducted during the lockdown (Cellini et al., 2020; Droit-Volet et al., 2020; Ogden, 2020; Torboli et al., 2020). However, the present studies investigating a large series of factors allowed us to identify the main causes of these changes in the subjective experience of time with the lockdown.

With regard to the living conditions during the lockdown (e.g., number of confined people, living space), contrary to what one might have thought, our studies showed that they had little or no significant influence on the sensation of time. Indeed, only in one study (Study 2) did time tend to drag when the living space was limited, whereas no such finding was observed in the other one (Study 1). Moreover, the results showed a significant but weak correlation between the available space and the level of boredom. Furthermore, the average area of the place of confinement for the participants was close in the second and the first study, and a significant effect was observed only in the former. The inconsistency of the results between the studies and the weakness of the effect suggests that it is not the space *per se* that really plays a role in the experience of time but what people do in this space and the emotion experienced by the person living in it. Writing, reading, and watching a good movie with your children all require little space. And one can be unhappy and bored on a large deserted island even if the sun is shining.

The participants reported multiple changes in their emotional states produced by the lockdown as well as by the associated context involving the spread of the virus. They were indeed overwhelmed by various negative emotions. They expressed fear and anxiety. They also described more anger and felt sadder and less happy than before the lockdown. Although most of these negative emotions are categorized as high-arousal emotions (Russell, 1980) (i.e., fear, anxiety, and anger), they were associated in our studies with a decrease in the level of arousal. The participants reported being calmer, more relaxed, and less stimulated/excited/awake during than before the lockdown. The general low arousal level described by the participants suggests that the emotions they reported corresponded more to a mood rather than to an emotion *per se*. Mood differs from emotion and is defined as an emotional state of moderate intensity (i.e., low arousal) that persists in time outside of the event or stimulus that triggered the emotion (e.g., Izard, 1991; Watson and Clark, 1994). In our studies, the participants thus reported an evaluative mental state that persisted over time during the lockdown period. The potential risk of the lockdown may therefore lie in the development of affective psychological disorders. It would be interesting to carry out a survey in a few months to check whether the disruption of affective states due to the Covid-19 crisis persists or not in order to evaluate the clinical consequences and to consider treatments and solutions. Nonetheless, the emotion scores assessed in our study remained quite low, being lower than 4 points on a seven-point rating scale. This suggests that the majority of our participants did not fall into an extreme negative affective state. Consistently with this, the considerable decrease in happiness that the participants experienced with the lockdown was not related to the increase in sadness, with this latter remaining low. Moreover, the effect of decreased happiness on the time judgment was not mediated by the increase in sadness. Nevertheless, our data indicated that the decrease in the feeling of being happy in the context of lockdown was greater in the participants with higher anxiety, depression, and impulsivity scores. But, the results of the two studies were not consistent for these two clinical dimensions. They suggest, however, that some people may suffer emotionally from the lockdown more than others due to their psychological vulnerability (Martinelli et al., unpublished).

Despite the upheaval of mood induced by the lockdown, our studies showed that few emotions directly affected the temporal experience. Indeed, the increase in the feeling of fear during lockdown was not associated with any changes in the subjective judgment of time. Similarly, the anxiety scores were not related to variations in the time judgment. And when a significant correlation with the time judgment was observed, it was mediated by other factors, namely boredom and decreased happiness (model 4, Figure 7). This is entirely consistent with the results of the study on time and Covid-19 conducted by Droit-Volet et al. (2020) showing that the time experience was not related to perceived stress about the virus and the disease or the perceived stress at home or work. In fact, the feeling of a slowing down of time during the lockdown was mainly related to the feeling of happiness. The happier people feel the faster time flies by. The participants felt unhappy during the lockdown and time therefore seemed to pass very slowly. This provides further evidence that the feeling of the PoT results from the participants’ introspective analyses of their internal emotional states (Droit-Volet et al., 2018b; Droit-Volet and Dambrun, 2019).

Our study therefore showed that the decrease in the individual levels of happiness explained the changes in the experience of time. However, these emotional changes did not constitute the main factor underlying the feeling of a slowing down of time during the lockdown. That factor was boredom. Indeed, the two studies presented in this manuscript systematically showed the significant relationship between boredom and the feeling of a slowing down of time. Our statistical mediation analyses indicated that the activity performed and the level of happiness mediated the effect of boredom on time judgment, although to a lesser extent in the case of the latter. Therefore, because individuals performed only a few activities that occupied their attention, they tended to get bored and time dragged on. This is consistent with the attentional model of timing (Zakay, 1989; Block and Zakay, 1996; Zakay and Block, 1996) and the results of numerous studies using a dual-task paradigm. According to these, time judgment directly depends on the amount of attention allocated to timing (Nobre and Coull, 2010). The more attentional resources the task being performed consumes, the more its duration is underestimated. Attentional mechanisms, related to the amount of activity that fills the time period to be estimated and the resulting feeling of boredom, would therefore underlie the subjective experience of time during lockdown. This provides support for Larson’s model of the critical role of occupational activity in the estimation of the speed of the PoT (Larson, 2004; Larson and von Eye, 2006). However, the emotion of happiness also played a significant role in the effect of boredom on the experience of time. Without further investigation, however, it is difficult to identify the mechanisms involved in the mediating role of happiness on the effect of boredom on the time judgment. It is likely that the feeling of happiness also involves attention mechanisms. For example, Droit-Volet et al. (2018a) showed that the practice of meditation exercises, when the participants were trained to focus their attention on different parts of their body (body scan) or breathing rhythm, both increased the feeling of happiness and produced an underestimation of time, with the feeling that time flies by. Consequently, the feeling of happiness *per se* may also depend on the orientation of the attentional focus toward activity, which in turn affects the time judgment. In sum, attention mechanisms could also be involved in the mediating effect of happiness on the boredom–time relationship.

Our studies showed that boredom was therefore the best predictor of feelings about the speed of time during lockdown. Although the concept of boredom has long been a subject of academic study, it is a complex emotion that has been neglected and under-investigated experimentally (Smith, 1981; Pekrun et al., 2010; van Hooft and van Hooff, 2018). The boredom-related processes that monitor temporal experiences therefore remain unclear (Zakay, 2014; Jokic et al., 2018; Witowska et al., 2020). Our study revealed that the effect of boredom on the temporal experience was partially mediated by the lack of activity and the decrease in happiness. However, our study also showed that boredom was not reduced to the effect of these two factors, as is indicated by the significant direct effect of boredom on the time judgment in the mediation models. Studies on perceptual deprivation have shown that humans need meaningful information (Merhabian, 1977). The lockdown situation could thus be also a poor and monotonous environment, as it is devoid of the successive events that usually fill the day and time.

The participants in our studies also reported that the rhythm of the days under lockdown was less regular than usual and, more importantly, that they found it more difficult to sleep during the lockdown than before it. Furthermore, it appears that sleep quality was significantly correlated with subjective time: The better people slept during the lockdown the faster time passed. What is more, our data suggested that sleep deregulation, related to the irregular life rhythm during the lockdown, was also a significant mediator of the effect of boredom on the slowing down of time during the lockdown. However, the significant sleep–time relationship was found in our second study but not in the first one. This can be explained by the fact that we tested two different populations in our study at slightly different moments during the lockdown, i.e., the latter for the second study. The link between sleep and PoT judgment must therefore be confirmed in future studies.

## Conclusion

During the lockdown imposed by the government due to the spread of Covid-19, the participants experienced a slowing down of time that was mainly explained by the sense of boredom which overwhelmed them, partly due to a lack of activity, some sleeping difficulties, and the relative negative feeling of being less happy. However, the participants reported that they had more time for themselves and that they were more calm and relaxed. They were nevertheless little focused on the present, i.e., with little propensity to concentrate on positive thoughts and bodily sensations related to the self (Fenigstein et al., 1975). It is possible that anxiety and uncertainty, especially about the time when lockdown would end, might have prevented them from focusing on the simple pleasures of the present moment and trying to find interesting activities. Being master of one’s time—that is, forgetting it and making it fly past—requires practice. The lockdown was too brutal. It was not prepared and organized by the people, and, in our industrial society, we have lost our autonomous control—our mastery of our time. We need time to conquer our time.

## Data Availability Statement

The raw data supporting the conclusions of this article will be made available by the authors, upon request.

## Ethics Statement

The studies involving human participants were reviewed and approved by the Research Ethics Committee of the University Clermont Auvergne (IRB00011540-2020-31). The ethics committee waived the requirement of written informed consent for participation.

## Author Contributions

NM, SG, CB, PH, and SD-V conceived the survey. NM collected the data, analyzed the data, and drafted the manuscript. SG and SD-V analyzed the data and drafted the manuscript. JC analyzed the data. CB, GD, JC, and PH provided critical revisions and approved the final version of the manuscript. PH and SD-V secured funding for the study. All authors contributed to the article and approved the submitted version.

## Conflict of Interest

The authors declare that the research was conducted in the absence of any commercial or financial relationships that could be construed as a potential conflict of interest.
